# Solubility and hydrolysis of Fe(ii) under hyperalkaline conditions

**DOI:** 10.1039/d6ra02690g

**Published:** 2026-07-06

**Authors:** Paul Quentin Fürst, Sven Hagemann, Neşe Çevirim-Papaioannou, Xavier Gaona, Marcus Altmaier, Horst Geckeis

**Affiliations:** a Karlsruhe Institute of Technology, Institute for Nuclear Waste Disposal (KIT-INE) Karlsruhe Germany p.fuerst@kit.edu xavier.gaona@kit.edu; b Gesellschaft für Anlagen- und Reaktorsicherheit Braunschweig Germany sven.hageman@grs.de

## Abstract

The speciation and solubility of iron under reducing, hyperalkaline conditions was investigated through undersaturation solubility experiments in combination with solid phase characterization by XRD. The solubility of a well-defined Fe(OH)_2_(cr) solid phase was studied at *T* = (25 ± 2) °C in aqueous solutions containing 0.10 to 4.20 m KOH and 1.06 to 22.73 m NaOH. Experimental data were used to derive chemical, thermodynamic and (Specific Ion Interaction Theory (SIT), Pitzer) activity models that accurately describe iron solubility under these conditions. These results support Fe(OH)_3_^−^ as the main aqueous iron(ii) species up to 1 m NaOH/KOH, while Fe(OH)_4_^2−^ dominates at higher hydroxide concentrations.

## Abstract 2

Iron(ii) solubility and speciation under reducing, hyperalkaline conditions are quantified using new experimental data and thermodynamic modeling approaches.

## Graphical abstract

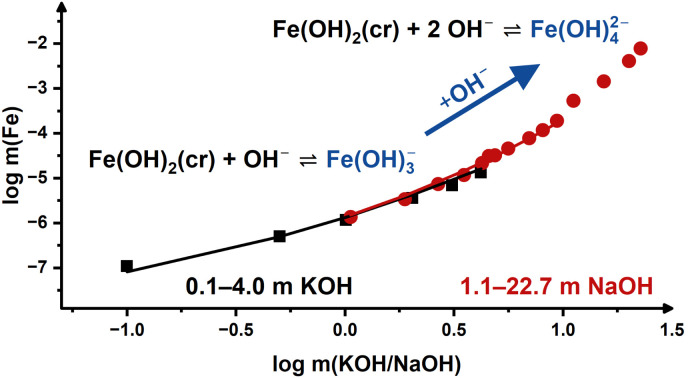

## Introduction

In reducing aqueous systems, iron commonly occurs in the oxidation state +II. Under alkaline conditions, Fe^2+^ is hydrolyzed according to the following reactions.^1^1
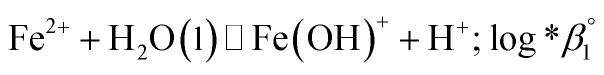
2
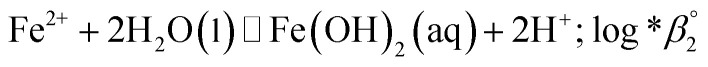
3
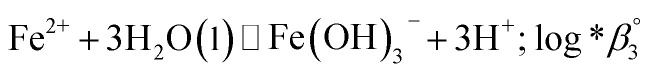


Reducing, hyperalkaline conditions are particularly relevant to the context of deep geological repositories, as they may arise from iron corrosion and cement degradation processes triggered by water intrusion.^2,3^ However, the available experimental data for Fe(ii) hydrolysis is limited, especially due to the experimental difficulties in stabilizing the oxidation state and the low iron concentrations, making potentiometric and spectrophotometric investigations challenging.^4–6^ While solubility experiments can be used to overcome these difficulties, they require the use of well-characterized solid phases which could not be provided by previous studies, as criticized within the comprehensive reviews of the Thermochemical Database Project by the Nuclear Energy Agency (NEA-TDB).^2,3^

The sole currently existing solubility study of iron(ii) hydroxide under hyperalkaline conditions was published in 1956 by Gayer and Woontner.^7^ The authors investigated the solubility of Fe(OH)_2_(s) in alkaline solutions at 25 °C and proposed the formation of two distinct hydrolysis species under hyperalkaline conditions: HFeO_2_^−^ (corresponding to Fe(OH)_3_^−^) and FeO_2_^2−^ (corresponding to Fe(OH)_4_^2−^). However, the solid phase used in this study was poorly characterized, relying solely on visual assessments of the color. Additionally, the procedures for activity corrections are not clearly described in the paper, which is particularly important given that systems containing up to approximately 6 M NaOH were examined. Furthermore, the methodologies for pH measurement and, in particular, for determining the base concentrations are not fully explained. The paper also does not address why the initial sodium hydroxide concentration differs from the equilibrium concentration by as much as one mole per liter.

Due to the limited data available on the solubility of Fe(OH)_2_(s) under hyperalkaline conditions, (1,3) and (1,4) Fe(ii) hydrolysis constants currently selected in main thermodynamic databases are primarily based on magnetite solubility data within the temperature range of 25 to 300 °C (see Table 1).^8–12^ Among these databases, ThermoChimie is the only one selecting the (1,4) hydrolysis species, *i.e.*, Fe(OH)_4_^2−^.

**Table 1:** Equilibrium constants reported and selected in the literature for iron(ii) hydrolysis under hyperalkaline conditions

Reaction	log *β°	Reference
Fe^2+^ + 3H_2_O(l) ⇌ Fe(OH)_3_^−^ + 3H^+^	−(33.84 ± 1.55)	TC 13a^5^
	−(32.68 ± 0.15)	PSI/Nagra^6^
	−(34.30 ± 0.20)	NEA-TDB^a^^2^
Fe^2+^ + 4H_2_O(l) ⇌ Fe(OH)_4_^2−^ + 4H^+^	−(46.32 ± 0.74)	TC 13a^5^

a Data discussed, but not selected in NEA-TDB.

The aim of this study is to clarify iron(ii) speciation under hyperalkaline conditions by investigating the solubility of a well-characterized solid phase, *i.e.*, Fe(OH)_2_(cr). Additionally, this work aims at providing complete and consistent thermodynamic and (SIT,^13^ Pitzer^14^) activity models for Fe(ii) valid in dilute to concentrated NaOH and KOH solutions. To allow for the reliable derivation of a chemical model, SIT and Pitzer parameters, a wide range of KOH and NaOH concentrations (up to 22.73 m NaOH) are selected in experiments. It is clear, that relevant hyperalkaline conditions in the cementitious environment of a deep geological repository are characterized by much lower KOH and NaOH concentrations.^15,16^

## Experimental

### Chemicals

To minimize the effect of atmospheric O_2_ and CO_2_ on the samples, all experiments were conducted in glove boxes under argon atmosphere (<0.1 ppm O_2_, *T* = (25 ± 2) °C). For the preparation of all samples and solutions, ultra-pure water, purified using a Milli-Q apparatus (Millipore, 18.2 MΩ, 25 ± 2 °C) and purged with argon gas for several hours, was used. KOH Titrisol© standard solution and concentrated NaOH (50 wt-% Emsure) used to prepare matrix solutions, were purchased from Merck. Sodium dithionite (Na_2_S_2_O_4_, tech., 85+%, Thermo Scientific) was added as reducing agent to selected samples. 2% HNO_3_ solution (prepared from 60% HNO_3_ Ultrapur, Merck) was used to dilute the samples prior to ICP-MS (Inductively Coupled Plasma Mass Spectrometry) analysis.

### Solid phase synthesis and characterization

Details on solid phase preparation, including chemicals used, and characterization (such as Rietveld analysis) can be found in Fürst *et al.*^17^ Briefly, Fe(OH)_2_(cr) was precipitated from a 1 M FeCl_2_ solution (prepared by dissolving iron powder in hydrochloric acid under argon atmosphere) with KOH solution and aged in 1 M KOH or 1 M NaOH solution over several months. X-ray diffraction (XRD, Bruker D8 Advance with Cu Kα X-ray source and LYNXEYE XE-T detector and Malvern Panalytical X Pert Pro MPD, Cu Kα X-ray source, X'Celerator detector) was used to characterize the initial solid phase.

### Solubility experiments

Undersaturation solubility experiments were conducted in 1.06 to 22.73 molal (*m*) (corresponding to 1 and 17 molar, respectively) NaOH and in 0.10 to 4.20 m (0.10 molar and 4.0 molar) KOH solutions.

Solubility measurements in NaOH were performed by addition of approximately 0.3 g of wet Fe(OH)_2_(cr) to about 13 g NaOH solution in 30 ml screw-capped NALGENE© HDPE bottles. The bottles were kept in dark boxes to avoid photooxidation. During several months, the batches were shaken every couple of days. Samples were taken after filtration through VWR© filters (0.22 µm, PTFE membrane, PP housing material). The hydroxide concentration was measured by potentiometric titration of weighed aliquots against a standardized HCl solution. Analogous experiments in KOH were performed both in the presence and absence of Na_2_S_2_O_4_ (5 mM) as reducing agent (15 ml screw-capped PP vials (Sarstedt); ultrafiltration using 10 kDa filters, pore size ≈ 2–3 nm, Nanosep, Pall Life Science). For the experiments conducted in KOH, the hydroxide concentration was assumed to be equal to the initial concentration of the base. Iron concentrations in solutions were regularly monitored for up to 258 days. ICP-MS (NexION 5000, PerkinElmer or 8900 ICP-QQQ, Agilent) was used to determine the aqueous iron concentration. The measurement uncertainty for Fe in NaOH solutions was ±10%. Densities of KOH solutions for conversion between molar and molal concentrations were adopted from Sipos *et al.*^18^ Due to gravimetric sampling analytical results for NaOH delivered molal concentrations without the need of solution densities. For KOH systems, redox potentials (*E*_h_) of selected samples were measured in suspension using a combined Pt and Ag/AgCl reference electrode (Metrohm).

## Results and discussion

### Solid phase stability

**Fig. 1:**
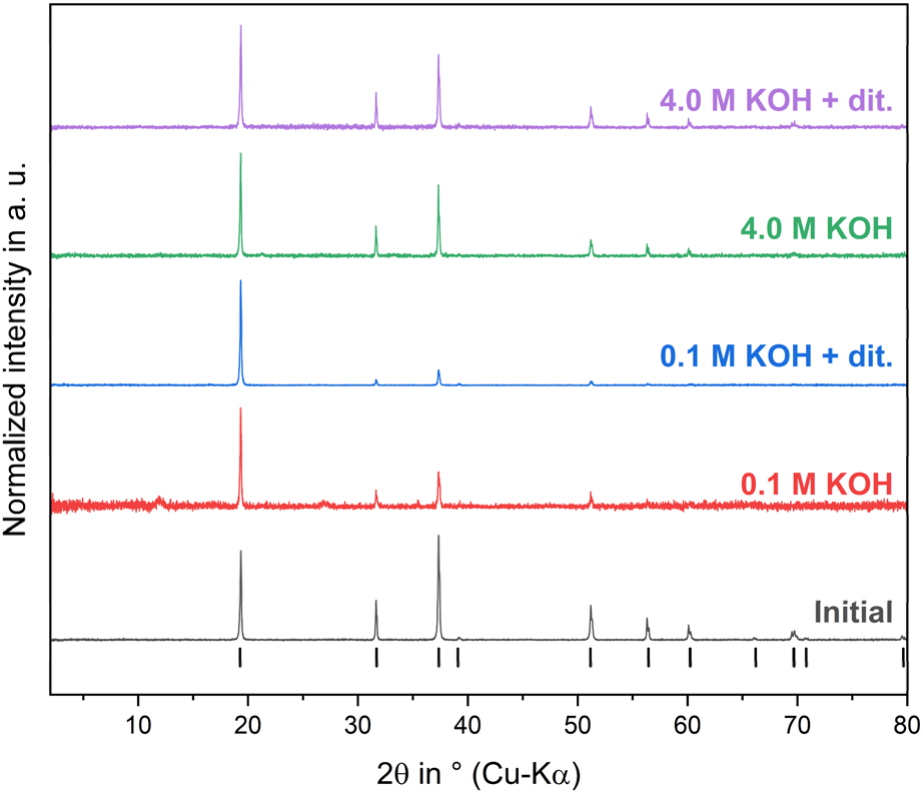
XRD spectra of Fe(OH)_2_(cr) initially and after reaching equilibrium within the solubility experiments with normalized intensities. Reference data for Fe(OH)_2_(cr) (COD 9009104 ^19^) shown as black dashes at the bottom.

Fe(OH)_2_(cr) was obtained as a white solid from synthesis. The initial precipitate sometimes contained tiny dark grains that did not change in quantity or size throughout the procedure or the later experiments. Drying the Fe(OH)_2_ under argon atmosphere in the glove box (O_2_ concentration below 0.1 ppm) always led to the formation of a very thin dark black–brown layer within a few hours. The amount of the dark products (grains, layer) was too small to be analyzed by XRD. When stored under 1 M NaOH, no color change was observed even after two years. After equilibration in the solubility experiments, slight color changes were observed in several samples of the KOH system. In the absence of Na_2_S_2_O_4_, most of the solids turned pale orange, partially, while especially in the presence of the reducing agent a light grey color was seen in some samples. Fig. 1 displays the XRD patterns of both the initial Fe(OH)_2_(cr) and selected solid phases collected after equilibration in KOH solutions. In all cases, the XRD spectra closely matched reference data for Fe(OH)_2_(cr) (COD 9009104 ^19^), confirming its identity as the predominant solid phase throughout the experiments.

Additional reflections at 2*θ* ≈ 12° and 27° were observed in the sample equilibrated in 0.1 M KOH. Although these could not be conclusively attributed to a specific phase, they may indicate the presence of trace amounts of Fe(iii)-containing (oxy)hydroxides formed by partial oxidation. The observed color changes further support the formation of minor Fe(iii) phases. This is not unexpected, given the small stability field of Fe(ii) under hyperalkaline conditions and the long equilibration times. Nonetheless, Fe(OH)_2_(cr) remained the dominant solid phase both in the absence and presence of the reducing agent and is expected to control the solubility in all samples as discussed further below.

### Iron solubility

**Fig. 2:**
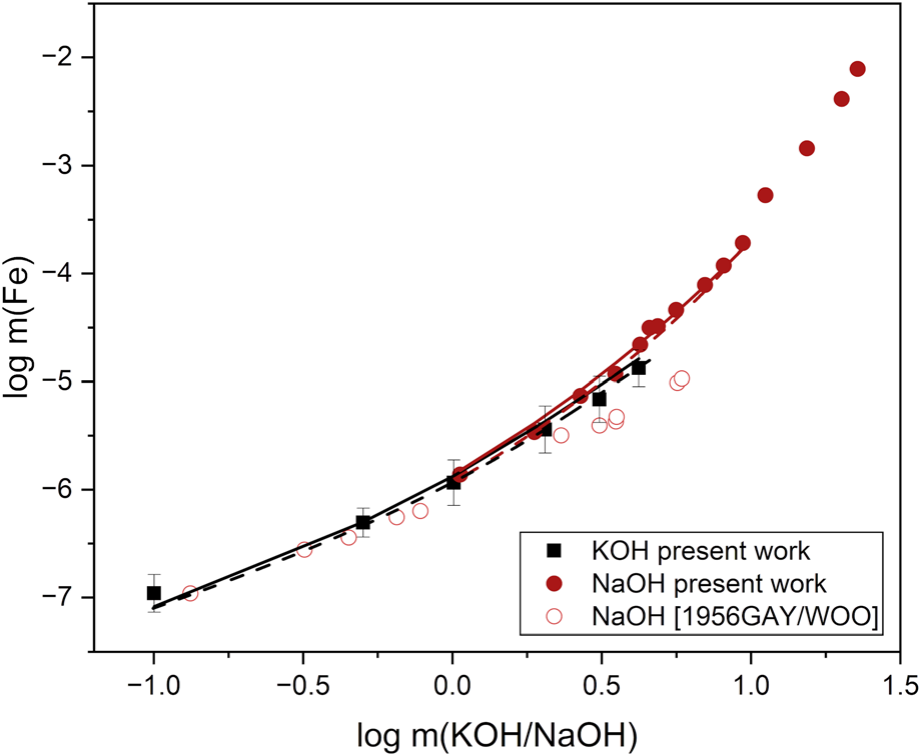
Iron solubility investigated in this work in NaOH (red filled circles) and KOH (black squares). The full experimental dataset is available from the SI (Tables S2 and S3). Data from Gayer and Woontner^7^ in NaOH (red hollow circles) is shown for comparison. Lines represent the SIT (solid) and Pitzer (dashed) models developed within this work.

Fig. 2 shows the measured aqueous iron concentrations (Tables S2 and S3) in KOH (black) and NaOH (red) solutions. For KOH, data are presented as mean value of up to four samplings, with uncertainties corresponding to the standard deviation of average values. No significant impact of the reducing agent was observed for any of the six ionic strengths (unpaired two-tailed *t*-test with Welch's correction, all *p* > 0.05, Table S4). *E*_h_ measurements of selected samples confirmed that reducing conditions were maintained throughout the solubility experiments (see Table S1 in the SI). This further supports the conclusion that trace oxidation of the solid phases, as suggested by minor color changes and XRD observations, has a negligible effect on the overall iron solubility. Red hollow circles represent data reported by Gayer and Woontner^7^ in NaOH, which fall within the uncertainty range of the measurements in KOH up to approximately 1 m hydroxide concentration, but deviate systematically at higher alkalinities. An increase in solubility is observed across the entire *m*(MOH) range investigated, with values of log *m*(Fe) rising from approximately −7.0 in 0.10 m KOH to −2.1 in 22.73 m NaOH solutions. Iron(ii) solubilities in more concentrated NaOH solutions differ slightly from those in KOH solutions, an observation familiar from other bivalent transition metals (*e.g.* Zn, Cd).^20^ On the logarithmic scale depicted in Fig. 2, the slope of this solubility increase grows with increasing hydroxide concentration, ranging from about +1 to as high as +4.

### Speciation

The speciation of Fe(ii) in alkaline solutions has long been considered to be similar to other M(ii) transition metal cations such as Co(ii), Cu(ii), or Mn(ii), assuming the formation of a full series of hydroxide complexes Fe(OH)_*n*_^2−*n*^ with *n* = 1–4.^21^ The assumption was based on Gayer and Woontner,^7^ the only study available at hydroxide concentrations above 1 m. It is notable that later reviews ignored this study and did not discuss the possible formation of Fe(OH)_4_^2−^ at all.^1,2^ Preliminary evaluations of our solubility data showed that the increase of iron solubility at hydroxide concentrations of 1 molal and higher can mathematically be described by a model that includes only Fe(OH)_3_^−^. However, the slope of the solubility curve reaching values of two and higher as well as highly unusual negative Pitzer ion interaction coefficients (*β*^(0)^ and *β*^(1)^) for the Fe(OH)_3_^−^ species were evidence that more than one hydroxide complex need to be considered in the system. There is no indication that higher complexes of Fe(ii) than the 1 : 4 complex exist at least up to 10 m, and most probably up to 22 m NaOH as will be later discussed.

### Thermodynamic modeling

To describe the experimental solubility data and to account for ionic strength effects, different activity models were developed. The Specific Ion Interaction Theory (SIT)^13^ is the method adopted for ionic strength corrections in NEA-TDB, among other thermodynamic databases. Besides the Debye–Hückel term,

4 *D* = 0.509 *I*_m_^0.5^/(1 + 1.5 *I*_m_^0.5^)

SIT introduces the ion interaction parameter *ε* for each ion pair of opposite charge to account for short-range, non-electrostatic interactions. SIT models were derived for NaOH and KOH systems assuming the prevalence of the solubility reactions (5) and (6), and imposing the same complexation constants at zero ionic strength for both electrolyte systems. For the development of the SIT model, the NaOH dataset was truncated at 10 m. Although this exceeds the typical application range for the SIT model, which is generally up to around 3.5 m,^2^ previous studies have demonstrated that the theory remains valid even at ionic strengths as high as 10 m, especially for 1 : 1 electrolytes.^22–27^ To account for the influence of ionic strength on the ion interaction parameter, an ionic strength-dependent formulation was adopted, as discussed below.

5 Fe(OH)_2_(cr) + OH^−^ ⇌ Fe(OH)_3_^−^

6 Fe(OH)_2_(cr) + 2OH^−^ ⇌ Fe(OH)_4_^2−^

7
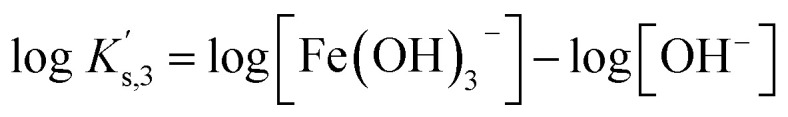
8
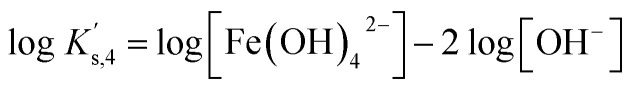


**Fig. 3:**
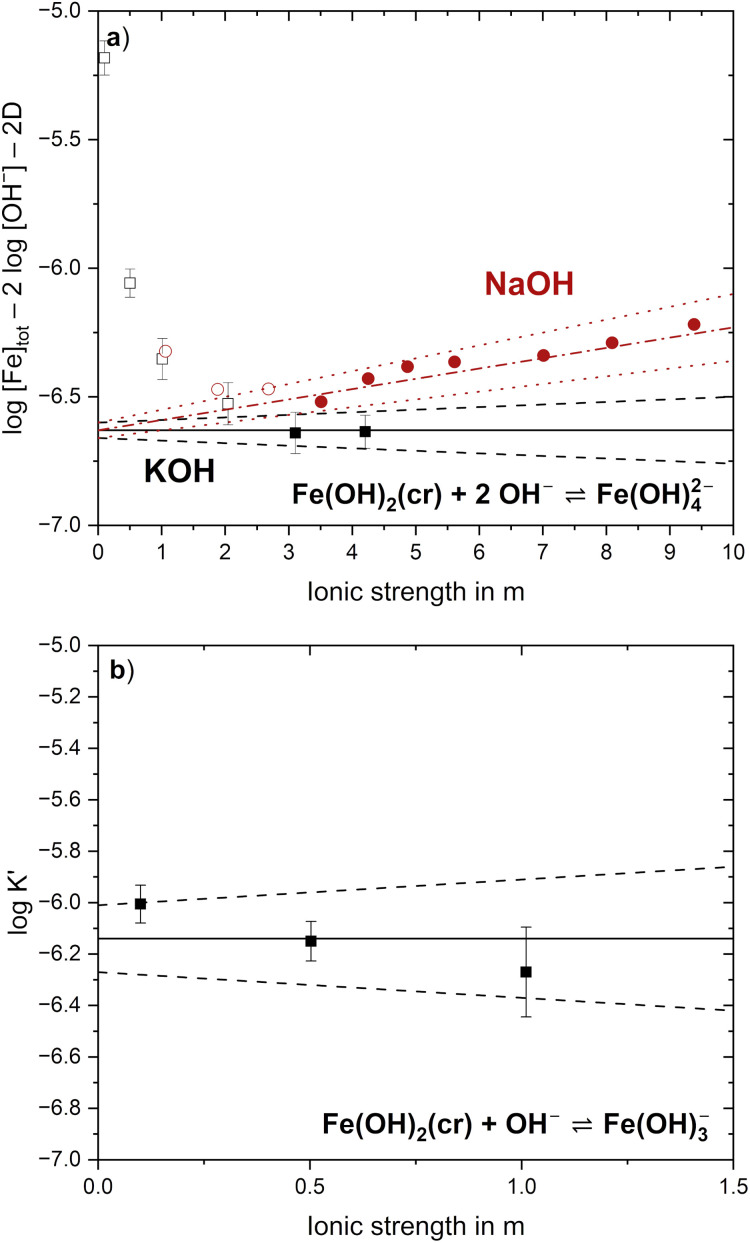
(a) SIT plot showing the correlation between log[Fe]_tot_ – 2 log[OH^−^] – 2D and the molal ionic strength in KOH (black squares, solid and dashed lines) and NaOH (red circles, dotted-dashed and dotted lines). Symbols represent experimental data, while lines represent linear regression (solid, dotted-dashed) and the corresponding uncertainty range extrapolated from zero ionic strength (dashed, dotted). Hollow symbols were not included in the linear regression since Fe(OH)_4_^2−^ is not the sole relevant hydrolysis species in this region. (b) SIT plot showing the correlation between the conditional log K′ and the molal ionic strength in KOH. [Fe(OH)_3_^−^] was calculated by the subtracting the calculated concentrations for the other relevant species, *i.e.*, [Fe(OH)_2_(aq)] and [Fe(OH)_4_^2−^] from the total experimentally determined iron concentration. Lines represent linear regression with a fixed slope (−Δ*ε* = 0.00 ± 0.1 kg mol^−1^) and the corresponding uncertainty range extrapolated from zero ionic strength.

Fig. 3a presents the SIT-plots for both electrolytes showing log[Fe]_tot_ – 2 log[OH^−^] – 2D according to eqn (6) and (8)*versus* the molal ionic strength defined by the concentration of the matrix solution. The matrix electrolyte concentration (NaOH or KOH) served as [OH^−^]. A clear linear trend is observed at ionic strengths above 3.0 m (filled symbols in Fig. 3a), indicating the dominance of the fourth hydrolysis species in this region. Here [Fe(OH)_4_^2−^] is assumed to be practically equal to the total iron concentration. There is no indication of further complexes up to at least 10 m NaOH. In contrast, data collected at lower ionic strengths deviate from this trend (hollow symbols in Fig. 3a), suggesting a substantial contribution from the third hydrolysis species under these conditions. Linear regression curves were simultaneously fitted to the experimental data at *I*_m_ > 3.0 m for both systems, with a shared *y*-axis intercept. Uncertainties are reported as the standard errors of the regression parameters. Ion interaction parameters and 
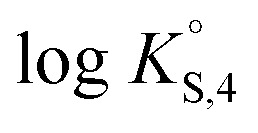
 values at zero ionic strength (Table 2) were obtained from the slope (−Δ*ε*(Na) = 0.04 ± 0.01 kg mol^−1^; −Δ*ε*(K) = −0.01 ± 0.01 kg mol^−1^), which corresponds to

9 −Δ*ε* = 2*ε*(Na^+^/K^+^, OH^−^) − *ε*(Na^+^/K^+^, Fe(OH)_4_^2−^)

**Table 2:** Solubility and hydrolysis coefficients as well as ion interaction coefficients (in kg mol^−1^) derived based on the experimental data collected in this work

Ion combination	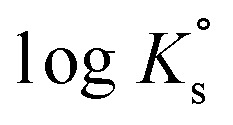	log *β°	*ε*_1_	*ε*_2_
Na^+^/Fe(OH)_3_^−^	−(6.16 ± 0.08)	−(32.48 ± 0.09)	0.011	0.021
K^+^/Fe(OH)_3_^−^	−(6.16 ± 0.08)	−(32.48 ± 0.09)	0.057	0.017
Na^+^/Fe(OH)_4_^2−^	−(6.60 ± 0.04)	−(46.92 ± 0.07)	−0.017	0.043
K^+^/Fe(OH)_4_^2−^	−(6.60 ± 0.04)	−(46.92 ± 0.07)	0.126	0.034
Na^+^/OH^−^a	—	—	0.011	0.021
K^+^/OH^−^a	—	—	0.057	0.017

a Derived from data compiled by Hamer and Wu^28^.

and the *y*-axis intercept. Ion interaction coefficients provided in NEA-TDB^2,3^ (*ε*(Na^+^, OH^−^) = (0.04 ± 0.01) kg mol^−1^; *ε*(K^+^, OH^−^) = (0.09 ± 0.01) kg mol^−1^) result in a significant deviation of the calculated activity coefficients (*γ*) at *I*_m_ > 2 m from experimental data compiled by Hamer and Wu^28^ as shown in Fig. S1 in the SI. Therefore, ionic strength dependent ion interaction parameters were derived according to the NEA-TDB guidelines^29^ from the experimental data following eqn (10).

10 *εI*_m_ = (*ε*_1_ + *ε*_2_*I*_m_^0.5^)*I*_m_

The derived ionic strength dependent ion interaction parameter can well describe the experimentally obtained activity coefficients even up to 10 m (*cf.* Fig. S1) and are presented in Table 2. Consequently, all SIT parameters (*ε*) derived in this work were obtained with an ionic strength dependency. The hydrolysis constant 
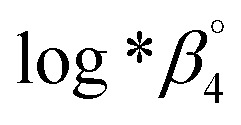
 was calculated combining the solubility constant 
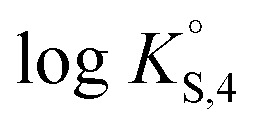
, the solubility product of Fe(OH)_2_(cr) as determined in our previous study (
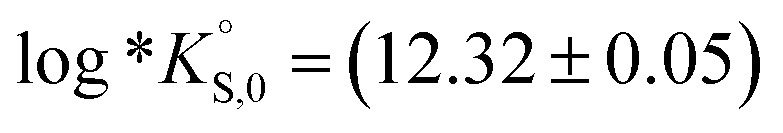
, Fürst *et al.*^17^) and the ionic product of water p*K*_w_.

Based on the solubility constant obtained for the fourth hydrolysis species 
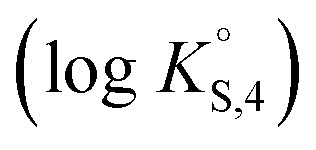
 and the complexation constant of the second hydrolysis species, as reported by Brown and Eckberg,^1^
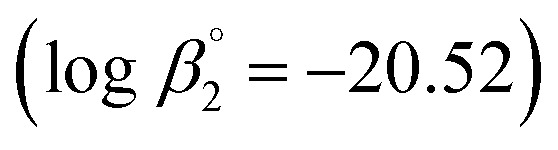
 the contribution of the third hydrolysis species was calculated from the measured total iron concentration [Fe]_tot_ according to eqn (11).

11 [Fe(OH)_3_^−^] = [Fe]_tot_ − [Fe(OH)_4_^2−^] − [Fe(OH)_2_(aq)]

**Fig. 4:**
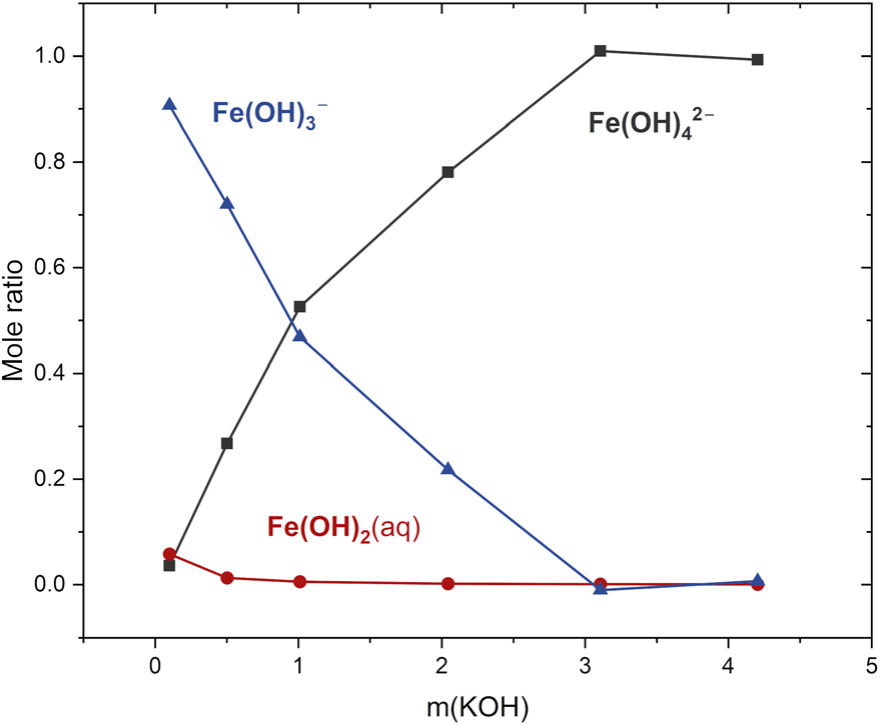
Aqueous iron speciation for the experiments in KOH. Fe(OH)_3_^−^ contribution was calculated from the measured total iron concentration and the calculated concentrations for Fe(OH)_2_(aq) and Fe(OH)_4_^2−^.

The resulting speciation diagram is presented in Fig. 4. This analysis demonstrates that the third hydrolysis species exhibits a maximum contribution of approximately 90% in 0.10 m KOH solution, which decreases to 47% at 1.01 m KOH. Consequently, the use of linear regression in the SIT plot becomes more challenging, as the slope is significantly affected by the varying contributions of other species. Nevertheless, a SIT plot was generated (Fig. 3b) by assuming a fixed slope of −Δ*ε* = 0.00 ± 0.1 kg mol^−1^, *i.e.*
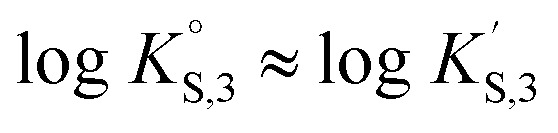
, which is reasonable for isocoulombic reactions, where ionic strength corrections are often minor.^2^ The results of this approach are summarized in Table 2.

The hydrolysis constant 
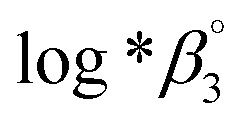
 determined in this work is 0.20 (PSI/Nagra^6^) to 1.33 (ThermoChimie^5^) log units higher than values currently selected in relevant thermodynamic databases and approximately 1.82 log units higher than the value discussed in NEA-TDB,^2^ indicating stronger hydrolysis than previously expected. Values reported (but not selected) in the NEA-TDB review considered only solubility experiments with Fe_3_O_4_(cr) at elevated temperatures (≥100 °C), thus requiring extrapolation over a wide temperature range to obtain the thermodynamic functions in the reference state. In line with the review work by Brown and Ekberg,^1^ the PSI/Nagra TDB selection by Hummel and Thoenen^6^ relied also in low temperature solubility data by Ziemniak and co-workers.^11^ We note that the latter authors assumed a solubility control by Fe(OH)_2_(cr) in systems below ≈ 120 °C, although no experimental confirmation of such solid phase was provided. The recent solubility study by Bruno *et al.* conducted with Fe_3_O_4_(cr) at *T* = (22 ± 2)°C resulted in 
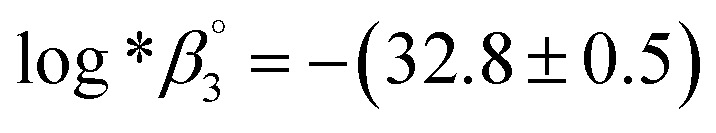
,^12^ in moderate agreement with the value determined in the present work. For the fourth hydrolysis constant, 
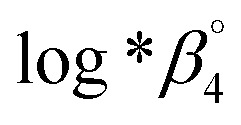
, the value derived in this work is approximately 0.6 log units lower than the current ThermoChimie^5^ selection; however, it still falls within the broad uncertainty range specified in the database (±0.74 log units).

Pitzer ion interaction coefficients were derived with the same experimental data set using the solubility constants (
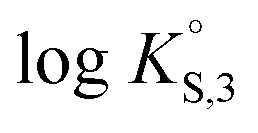
 and 
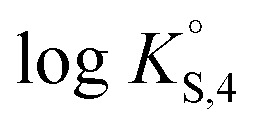
) previously determined by the SIT evaluation. In the first step, data points were selected where Fe(OH)_4_^2−^ is practically the only Fe(ii) species present (>3 m MOH). However, this limits the range of usable data and makes a proper determination of *β*^(1)^ for M^+^/Fe(OH)_4_^2−^ difficult. Therefore, data at lower MOH concentrations were also included by using the approximated free concentration of Fe(OH)_4_^2−^ at *c*_MOH_ between 1 and 3 m (as shown in Fig. 4). Solubility data above 10 m NaOH were not considered as the Pitzer model for NaOH used in this study (THEREDA release 2023a^30,31^) was derived only for solutions below this limit.

Several combinations of interaction parameters were tested, and the set shown in Table 3 provided the best results. At all MOH concentrations, the complex Fe(OH)_3_^−^ exists in equilibrium with significant amounts of either Fe(OH)_2_(aq) or Fe(OH)_4_^2−^. The free concentrations of Fe(OH)_3_^−^ in the concentration range 0.1 to 1.1 m MOH were estimated using the SIT model and interaction parameters derived that could describe the equilibrium with Fe(OH)_2_(cr). Data for NaOH and KOH solutions were combined in a single data set, assuming that interaction coefficients with Na^+^ and K^+^ are the same within the limited concentration range.

**Table 3:** Pitzer ion interaction coefficients for Fe(OH)_3_^−^ and Fe(OH)_4_^2−^ in NaOH and KOH medium derived from the experimental data collected in this work

Ion combination	*β* ^(0)^	*β* ^(1)^ (*α*_1_ = 2)	*C* ^*φ*^	Max *c* (MOH) [*m*]
Na^+^/Fe(OH)_3_^−^	0.359	0	0	1
K^+^/Fe(OH)_3_^−^	0.359	0	0	1
Na^+^/Fe(OH)_4_^2−^	0.311	1.068	−0.0140	9.4
K^+^/Fe(OH)_4_^2−^	0.384	1.796	0	4.2
Na^+^/OH^−^a	0.104	0.125	0.0022	10
K^+^/OH^−^a	0.137	0.335	0.0018	5

a Data from the THEREDA database release 2023a^30,31^

Due to the low number of available data points and the limited range of NaOH/KOH concentrations where the complex Fe(OH)_3_^−^ prevails, it was considered more robust to derive only a value for *β*^(0)^. Setting a fixed value for *β*^(1)^ (*e.g.*, to the mean value for 1–1 systems in Humphreys *et al.*^32^) did not lead to better results.

Additional coefficients were tested but did not result in significantly improved representation of observed solubilities. Using this model, the calculated solubilities of Fe(OH)_2_(cr) in NaOH and KOH media correspond very well with the experimental data. The model works well even at concentrations of 15 m NaOH which is beyond the application limit of the selected Pitzer model for NaOH (Fig. S2). As the solubility curve up to 22.7 m NaOH shows no new features, we assume that no further iron(ii) hydroxide species are formed.

## Summary and conclusions

In this study, the solubility of a well-characterized Fe(OH)_2_(cr) solid phase was investigated under hyperalkaline conditions in both sodium and potassium hydroxide solutions. Reducing conditions were maintained throughout the experimental period of up to 258 days, even without the addition of any reducing agent. Both Fe(OH)_3_^−^ and Fe(OH)_4_^2−^ were shown to contribute to the total iron concentration. Chemical, thermodynamic and (SIT, Pitzer) activity models developed from the experimental data accurately predict iron concentration under the examined conditions.

## Author contributions

P. Q. Fürst: writing – review & editing, writing – original draft, methodology, investigation, formal analysis. S. Hagemann: writing – review & editing, writing – original draft, investigation, methodology, project administration, funding acquisition, conceptualization. N. Çevirim-Papaioannou: writing – review & editing, supervision, methodology. X. Gaona: writing – review & editing, supervision, project administration, funding acquisition, conceptualization. M. Altmaier: writing – review & editing, project administration, funding acquisition, conceptualization. H. Geckeis: writing – review & editing, supervision.

## Conflicts of interest

There are no conflicts to declare.

## Supplementary Material

RA-OLF-D6RA02690G-s001

## Data Availability

The data supporting this article have been included as part of the supplementary information (SI). Supplementary information is available. See DOI: https://doi.org/10.1039/d6ra02690g.
