# Supplementary material for: Solubility and hydrolysis of Fe(ii) under hyperalkaline conditions
Source: RSC Adv. 2026 Jul 6;16(35):36843–9. doi: 10.1039/d6ra02690g (PMC13334385; doi:10.1039/d6ra02690g)
Supplement: RA-016-D6RA02690G-s001 [file RA-016-D6RA02690G-s001.pdf]

Supplementary Information (SI) for RSC Advances.
This journal is © The Royal Society of Chemistry 2026

Supplementary information

Table S1 E_h measurements of selected samples in the KOH system. Typical uncertainties in E_h measurements: ± 15 mV.

**M(KOH)**	**E_h in mV**
**Absence of $Na_2S_2O_4$, 84 days**	
0.1	-632.6
0.5	-693.2
1.0	-720.9
2.0	-749.7
3.0	-748.7
4.0	-749.2
**Presence of $Na_2S_2O_4$, initial**	
0.1	-616.3
0.5	-718.4
1.0	-838.8
2.0	-900.4
3.0	-908.5
4.0	-940.1

Table S2 Iron concentration (molal) determined in NaOH solutions. Errors in NaOH amount to ± 3%, measured Fe concentration have typical uncertainty of ± 10%.

NaOH [mol/kg]	$Fe(OH)_2$ [mol/kg]
1.06	1.38E-06
1.88	3.42E-06
2.68	7.40E-06
3.51	1.18E-05
4.25	2.19E-05
4.57	3.14E-05
4.87	3.26E-05
5.61	4.61E-05
7.01	7.84E-05
8.09	1.19E-04
9.38	1.92E-04
11.16	5.34E-04
15.37	1.45E-03
20.10	4.12E-03
22.73	7.83E-03

Table S3 Iron concentrations (molar) determined in KOH solutions. The datapoint marked with an asterisk (*) was identified as an outlier according to the 1.5 × IQR criterion (i.e., values lying more than 1.5 times the interquartile range above the third quartile or below the first quartile) and was therefore removed from the dataset. Analytical uncertainty typically ± 1%.

without reducing agent — all data in mol/l

M(KOH)	M(Fe) 5 days	M(Fe) 69 days	M(Fe) 83 days	M(Fe) 258 days
4.0	1.80E-05	5.60E-06	6.48E-06	1.24E-05
3.0	1.08E-05	1.94E-06	2.41E-06	7.70E-06
2.0	5.49E-06	1.17E-06	1.02E-06	4.34E-06
1.0	1.51E-06	4.74E-07	6.38E-07	1.04E-06
0.5	7.55E-07	3.05E-07	4.09E-07	4.54E-07
0.1	1.42E-07	7.18E-08	7.05E-08	5.51E-08

with reducing agent ($Na_2S_2O_4$) — all data in mol/l

M(KOH)	M(Fe) 9 days	M(Fe) 23 days	M(Fe) 198 days
4.0	1.45E-05	1.74E-05	1.41E-05
3.0	7.58E-06	8.09E-06	7.81E-06
2.0	4.82E-06	4.29E-06	3.55E-06
1.0	1.25E-06	2.13E-06	9.82E-07
0.5	4.84E-07	5.44E-07	*2.09E-06
0.1	1.74E-07	1.20E-07	1.29E-07

Table S4 Welch's unpaired two-tailed t-test statistics for the effect of reducing agent on aqueous iron concentration at different KOH concentrations. No comparisons reached statistical significance (all $p > 0.05$).

M(KOH)	t-statistic	degrees of freedom	p
4.0	-1.54	3.73	0.20
3.0	-0.99	3.03	0.40
2.0	-1.02	3.61	0.37
1.0	-1.29	3.69	0.27
0.5	-0.33	3.51	0.76
0.1	-2.19	4.99	0.08

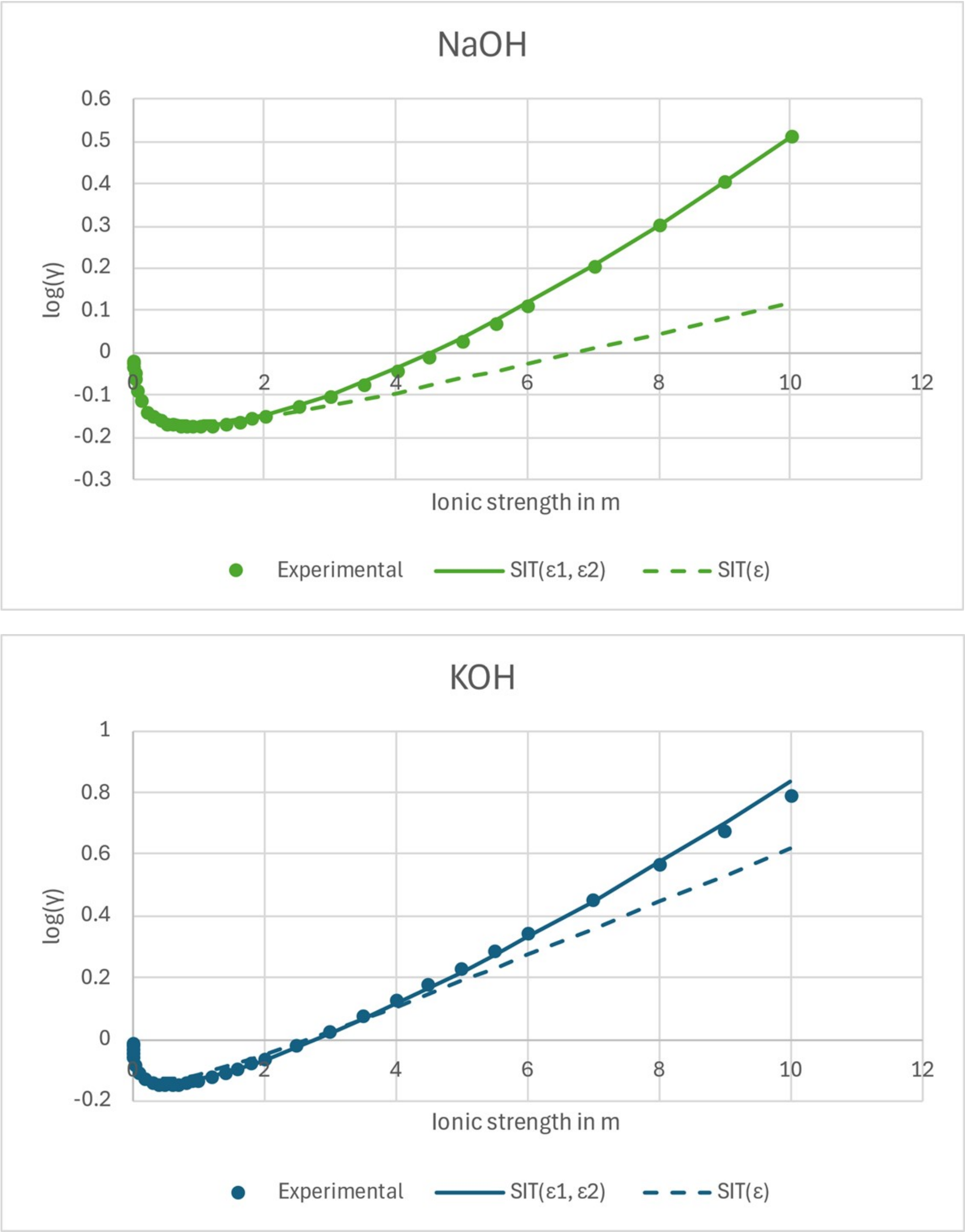

Figure S1 Comparison of experimentally obtained mean activity coefficients[28] for the M^+/OH^- ion pair compared to calculated data using either the classical SIT approach with one ion interaction parameter according to NEA-TDB[2,3] selection (dashed lines) or an ionic strength dependent ion interaction coefficient (solid line) derived according to the NEA-TDB guidelines[29] based on the experimental data compiled by Hamer and Wu.[28]

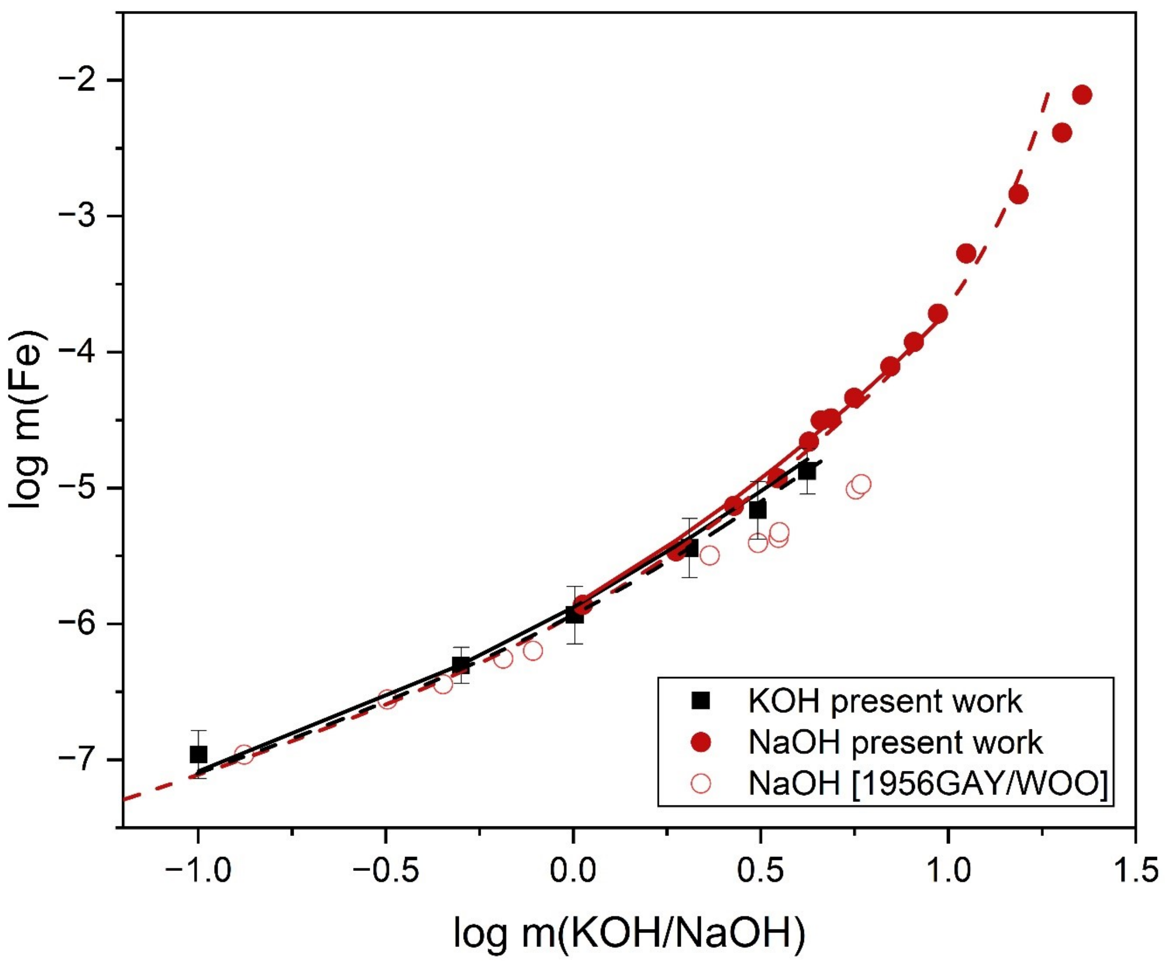

Figure S2 Extended Figure 2 showing the calculated solubility in NaOH beyond the application limit of the Pitzer model (10 m).
